# High-resolution ultrasound tendon-to-bone distances in partial and complete finger flexor A2 pulley ruptures simulated in human cadaver dissection: toward understanding imaging of partial pulley ruptures

**DOI:** 10.3389/fbioe.2023.1123857

**Published:** 2023-06-07

**Authors:** Xeber Iruretagoiena, Volker Schöffl, Ramón Balius, Marc Blasi, Fernando Dávila, Xavier Sala, Igor Sancho, Javier De La Fuente

**Affiliations:** ^1^ Deusto Physical TherapIker, Physical Therapy Department, Faculty of Health Sciences, University of Deusto, San Sebastián, Spain; ^2^ Eskura Osasun Zentroa, Beasain, Spain; ^3^ Sputnik Investigación, Madrid, Spain; ^4^ Section Sportsorthopedics and Sportsmedicine, Department of Orthopedic and Trauma Surgery, Klinikum Bamberg, Bamberg, Germany; ^5^ Department of Trauma Surgery, Friedrich Alexander University of Erlangen-Nuremberg, Erlangen, Germany; ^6^ Section of Wilderness Medicine, Department of Emergency Medicine, University of Colorado School of Medicine, Denver, CO, United States; ^7^ School of Clinical and Applied Sciences, Leeds Becket University, Leeds, United Kingdom; ^8^ Consell Catala de l´Esport, Generalitat de Catalunya, Barcelona, Spain; ^9^ Sport Medicine and Imaging Department, Clínica Diagonal, Barcelona, Spain; ^10^ Department of Plastic Surgery, Hospital Germans Trias I Pujol, Barcelona, Spain; ^11^ Orthopedics Department, Hospital Bidasoa, Irun, Spain; ^12^ Anatomy and Embryology Department, School of Medicine, Universitat de Barcelona, Barcelona, Spain; ^13^ Department of Anesthesiology, Hospital Clínic de Barcelona, Barcelona, Spain; ^14^ Orthopedics Department, Clínica Pakea-Mutualía, San Sebastián, Spain

**Keywords:** A2 pulley, tear, climbing, partial rupture, ultrasound, tendon-bone distance

## Abstract

**Introduction:** The A2 pulley tear is the most common injury in rock climbing. Whereas complete A2 pulley ruptures have been extensively researched, studies focused on partial A2 pulley ruptures are lacking. A2 pulleys rupture distally to proximally. High-resolution ultrasound imaging is considered the gold-standard tool for diagnosis and the most relevant ultrasound measurement is the tendon-to-bone distance (TBD), which increases when the pulley ruptures. The purpose of this study was to establish tendon-to-bone distance values for different sizes of partial A2 pulley ruptures and compare these values with those of complete ruptures.

**Material and methods:** The sample consisted of 30 *in vitro* fingers randomly assigned to 5 groups: G1, no simulated tear (control); G2, simulated 5 mm tear (low-grade partial rupture); G3, simulated 10 mm tear (medium-grade partial rupture); G4, simulated 15 mm tear (high-grade partial rupture); and G5, simulated 20 mm or equivalent tear (complete rupture). A highly experienced sonographer blinded to the randomization process and dissections examined all fingers.

**Results:** The tendon-to-bone distance measurements (medians and interquartile ranges) were as follows: G1, 0.95 mm (0.77–1.33); G2, 2.11 mm (1.78–2.33); G3, 2.28 mm (1.95–2.42); G4, 3.06 mm (2.79–3.28); and G5, 3.66 mm (3.55–4.76). Significant differences were found between non-torn pulleys and simulated partial and complete pulley ruptures.

**Discussion:** In contrast, and inconsistent with other findings, no significant differences were found among the different partial rupture groups. In conclusion, the longer the partial pulley rupture, the higher the tendon-to-bone distance value. The literature is inconsistent regarding the tendon-to-bone distance threshold to diagnose a partial A2 pulley rupture. The minimum tendon-to-bone distance value for a partial rupture was 1.6 mm, and tendon-to-bone distance values above 3 mm suggest a high-grade partial pulley rupture (15 mm incision) or a complete pulley rupture.

## 1 Introduction

Rupture of the finger flexor A2 pulley is the most common injury in rock climbers (Miro et al., 2021). The ring finger is most frequently affected, followed by the middle finger (Bollen, 1988). Pulley ruptures account for up to 33% of all rock climbing injuries (King and Lien, 2017). Climbing is rapidly gaining popularity, as reflected by its debut in the Olympics and a steep rise in climbing sport federation members (Lutter et al., 2017). As a result of this sport’s growth and development, the frequency of climbing-related pulley injuries is increasing (Miro et al., 2021).

The annular pulleys are the retinaculum portions that form part of the fibro osseous sheath containing the tendons of the muscles flexor digitorum superficialis (FDS) and flexor digitorum profundus (FDP) at the finger level (Martinoli et al., 2005). Their main role is to hold these tendons close against the phalanges to optimize their biomechanical function and avoid a bowstring deformity (Lin et al., 1990; Schöffl et al., 2017). Of the five annular and three cruciform pulleys, with the A2 pulley is considered the most important (Doyle, 2001). The A2 pulley inserts into the periosteum of the proximal phalanx on both sides, encircling the anterior aspects of the FDS and FDP tendons. Cadaver measurements confirmed that it is the longest of the pulleys, varying from 16.8 mm (Doyle, 1988) to 20 mm (Moutet, 2003). The length of the A2 pulley varies between fingers; the order from longer to shorter is the middle finger (16.4–20.5 mm), then ring (15.1–18.9 mm), index (12.8–15.9 mm), and little (11.7 mm) finger (Doyle, 1988; Schöffl et al., 2017). It spans the proximal and middle third of the proximal phalanx. From the base of the middle phalanx, its proximal margin is located at 30.6 mm and its distal rim at 15.5 mm (Moutet, 2003). The thickness ranges from 0.3 (Martinoli et al., 2000) to 0.7 mm (Schreiber et al., 2015), and it tends to be thicker in climbers (1.2 mm) (Klauser et al., 2000). The A2 is the strongest of the finger pulleys (Schöffl et al., 2009b).

Annular pulley ruptures may be complete or partial (Mitsionis et al., 2000). As a physical examination is non-specific, imaging tests are needed to determine the grade of injury and the number and extent of injured pulleys (Iruretagoiena-Urbieta et al., 2020a). Ultrasound is considered the diagnostic procedure of choice, as it allows for both static and dynamic assessment (Schöffl et al., 2017). The most relevant ultrasound finding is an increased distance of the flexor tendons to the palmar aspect of the base of the phalanx during dynamic examination maneuvers, along with peritendinous fluid (Martinoli et al., 2005). This distance is referred to as the tendon-to-bone distance (TBD) (Klauser et al., 2002).

A complete A2 pulley rupture leads to a significant increase in the TBD, decreased strength in finger flexion (Iruretagoiena-Urbieta et al., 2020b), and reduced range of motion of the proximal interphalangeal joint (PIP) (Bowers et al., 1994). However, the heterogeneity of ultrasound examinations (probe frequency, coupling agent, anatomical landmark, finger position, and load) (Iruretagoiena-Urbieta et al., 2020b) means that the ultrasound TBD cutoffs that define a complete A2 pulley rupture vary among the different publications, namely, from 1.9 mm (Schöffl et al., 2017) to 5.1 mm (Bodner et al., 1999). Most researchers have used a 2 mm threshold to diagnose a complete pulley rupture (Miro et al., 2021).

Although complete A2 pulley ruptures have been extensively investigated, studies describing its partial rupture are scarce (Iruretagoiena-Urbieta et al., 2020b). Such ruptures begin from distal to proximal (Hauger et al., 2000) as a consequence of friction between the pulley and flexor tendon and eccentric stress (Schöffl et al., 2009a). A large partial rupture, spanning close to 75% of the total pulley length, is associated with a reduced capacity to tolerate the traction force of the flexor tendon against the pulley (Mitsionis et al., 2000), along with a slight reduction in the PIP range of motion (Mitsionis et al., 1999). Overall ultrasound is the most valuable diagnostic tool for pulley ruptures (Klauser et al., 2002); for the ultrasound diagnosis of a partial rupture, little evidence and much controversy exist over reference TBD values (Iruretagoiena-Urbieta et al., 2020b). The literature provides different cut offs for the diagnosis of partial ruptures, ranging from TBD values from >2.2 mm (Hauger et al., 2000) to <2 mm (Schöffl et al., 2003), which may even coincide with those indicated for complete ruptures (Bodner et al., 1999).

In a proposed classification system for A2 pulley lesions in climbers, four grades are defined: sprain, partial rupture, complete rupture, and multiple ruptures (Schöffl et al., 2003). Partial ruptures of the A2 pulley are graded as a grade 2 lesion and are associated with an estimated recovery time period (RTP) of 8–10 weeks (Lutter et al., 2021). In contrast, an isolated complete A2 rupture is described as a grade III lesion with an estimated RTP of 3 months (Lutter et al., 2021). Thus, distinguishing between a partial and complete rupture is crucial to plan exact patient management, time to recovery, and accurately determine prognosis.

The main aim of this study was to establish TBD values for partial A2 pulley ruptures compared with those of complete ruptures. We also sought to examine whether a higher grade of simulated partial rupture leads to an increase in TBD possibly contributing to the disparate values reported in the literature, sometimes even overlapping those proposed for complete tears.

## 2 Materials and methods

This was a cross-sectional study conducted on human cadavers. Partial and complete A2 pulley ruptures were simulated through surgical incision and evaluated with ultrasound. A total of 30 fingers (10 index, 10 middle, and 10 ring fingers) from 5 fresh frozen human cadaver arms (average age 78 years, range 75–82 years) were studied. The little fingers were excluded due to anatomic and biomechanical reasons. The specimens had no signs or history of finger, hand, or wrist injuries or surgery and were left to thaw at room temperature before dissection. All specimens were obtained from bodies donated to the Faculty of Medicine and Health Sciences (Clinic Campus) of the University of Barcelona. Institutional review board approval was obtained prior to the study. The used cadaver tissues were part of a body donation program and in compliance with current Spanish legislation about ethics in research. None of the specimens showed trauma, deformities, or surgical scars on the hand.

**FIGURE 1:**
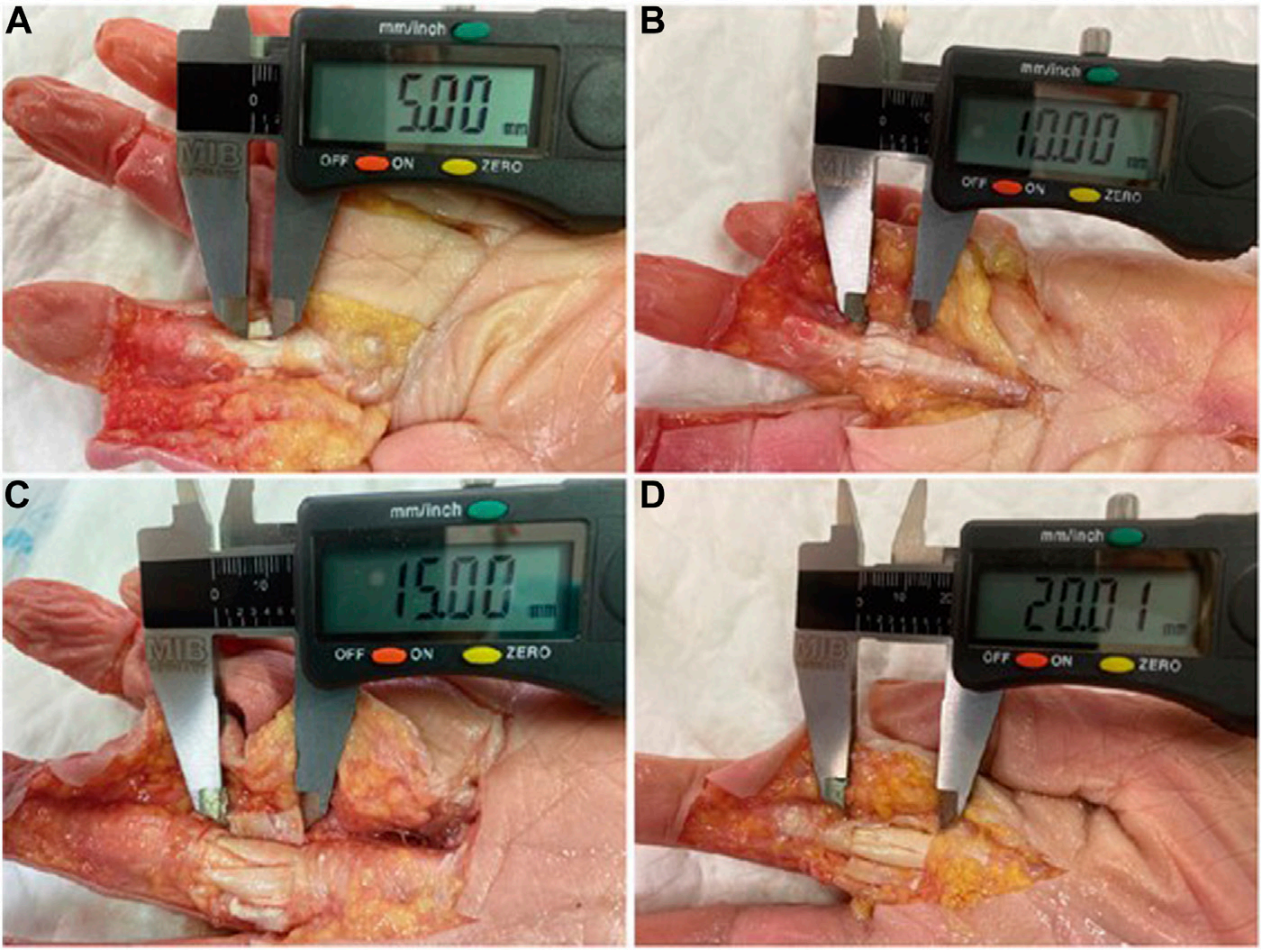
Simulated A2 pulley tears measurement: G2 **(A)**; G3 **(B)**; G4 **(C)**; G5 **(D)**.

All fingers were initially dissected by performing a single unilateral longitudinal incision at the transition between the dorsal and palmar skin from the metacarpophalangeal (MCP) joint to the distal interphalangeal (DIP) joint. Then, the subcutaneous fat layer to the finger pulley system was dissected without disrupting it. All fingers were randomly assigned to one of the following injury simulation groups: G1, no simulated tear (control); G2, simulated 5 mm tear (low-grade partial rupture); G3, simulated 10 mm tear (medium-grade partial rupture); G4, simulated 15 mm tear (high-grade partial rupture); and G5, simulated 20 mm or equivalent tear (complete rupture) (Figure 1). Prior to sectioning the pulleys from distal to proximal on their volar aspect, the distances to be incised according to the group assignment were measured with a digital caliper (Qfun ^®^ digital caliper, China, 0–150 mm, CN). After proper processing, abundant ultrasound gel was placed over the whole pulley system, which was then again covered with the previously raised skin and subcutaneous flap.

**FIGURE 2:**
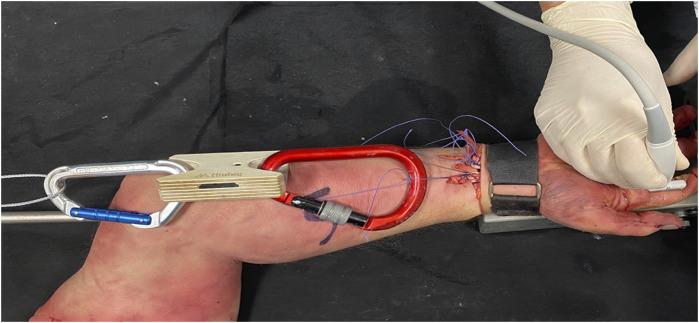
Finger flexor tendon tension system.

In addition, before ultrasound examination, the FDS and FDP tendons were exposed with a transverse incision at the forearm, proximal to the flexor retinaculum. Then, the FDS and FDP tendons in each finger were identified, and both flexor tendons corresponding to each finger were sutured using a polyglactin thread (Vicryl 2 ^®^, Ethicon, United States). To simulate flexor tendon tension, the sutured flexor tendons were isolated with a screw-locking carabiner clip and attached using a rigid aluminum wire to a Tindeq force sensory system (Tindeq^®^, sampling frequency: 80 Hz, design load: 150 kg, NO) (Figure 2).

**FIGURE 3:**
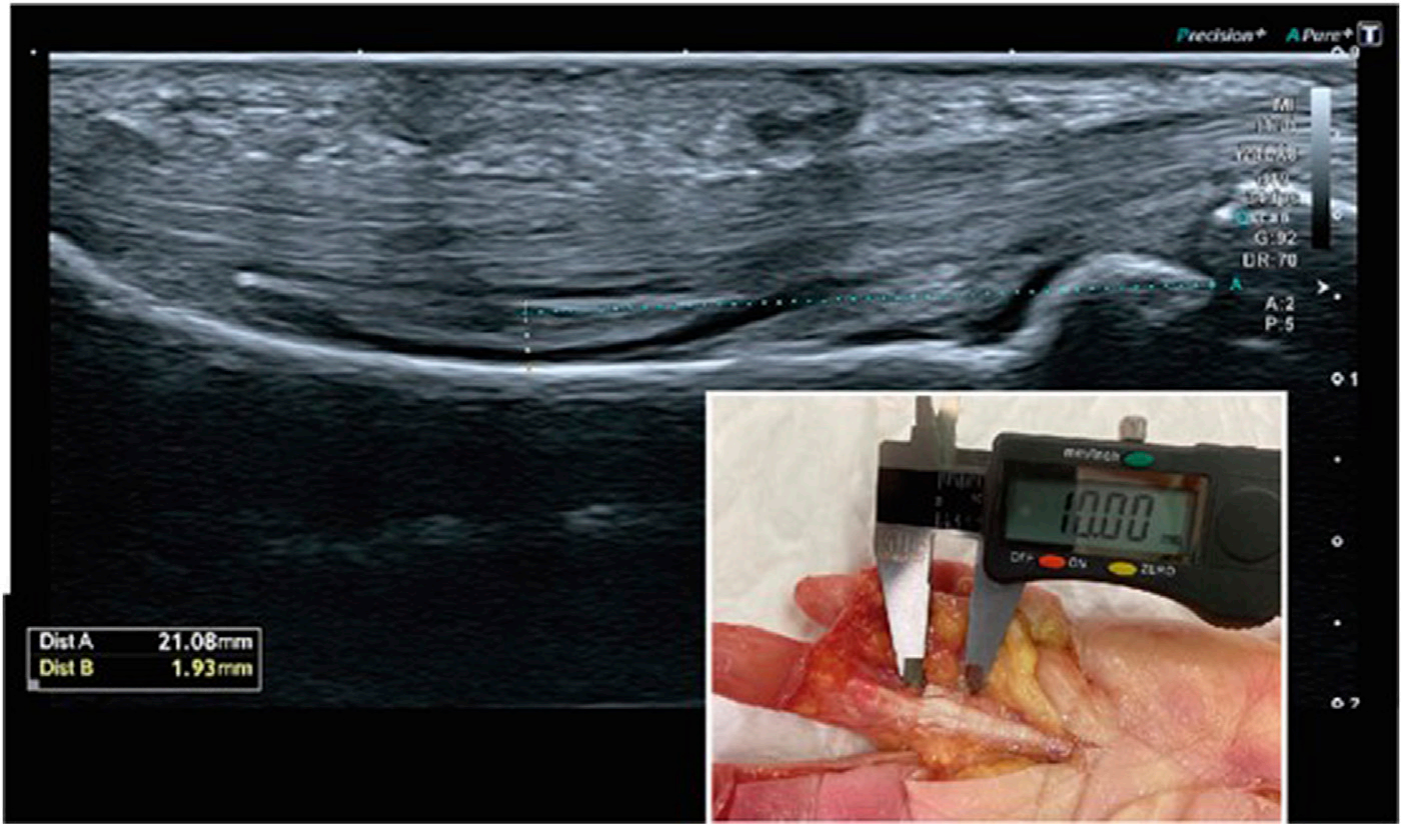
US TBD (1.9 mm) of a simulated 10 mm A2 pulley tear.

For ultrasound examination, we used a Canon Aplio i800 ultrasound machine equipped with a 22 MHz ultra-high-frequency hockey stick (i22LH8) and a 24 MHz ultra-high frequency iDMS linear transducer (i24LX8) (Canon medical system ^®^, United States). A single sonographer (JDF, with over 25 years of experience in musculoskeletal ultrasound), blinded to the previous randomization process and dissections, examined and measured all fingers. Abundant ultrasound gel was used to avoid compression of the finger by the transducer. The finger examination position was 0° or neutral MCP joint, 40° of flexion of the PIP joint and 10° of flexion of the DIP joint with a constant traction force of 5 kg directly applied to the FDS and FDP tendons. First, the proximal phalanx was measured using a linear transducer to estimate the midpoint of the phalanx. Once this anatomic landmark was located, a stick transducer was used to measure the TBD at the level of the midpoint of the proximal phalanx (Figure 3).

Data are described using the most appropriate statistics for the nature and scale of measurement of each variable: absolute and relative frequencies in percentages, mean and standard deviation for continuous variables, and median and interquartile range when appropriate according to the data distribution. The data are graphically represented through box plots. For quantitative variables, the Shapiro–Wilk test was used to check normality. To compare TDB means, we used ANOVA and Bonferroni correction for 2-by-2 pairwise comparisons of the different length sections incisions of the A2 pulleys. Due to the small sample size for each of the A2 pulley sections, the corresponding non-parametric test (Wilcoxon test) was employed along with the Jonckheere–Terpstra trend test. Correlations between variables were assessed with Spearman’s correlation. The software used for data analysis was Stata SE for Windows (Stata Corp ^®^. 2021. Stata Statistical Software: Release 16. College Station, TX: StataCorp LLC, United States). Significance was set at *p* < 0.05.

## 3 Results

Of the 30 initial fingers included, 6 could not be examined as too much air had accumulated between the dissected tissues despite the abundant gel applied between the pulley system and the overlying dissected skin and subcutaneous flap.

For a final study sample of A2 pulleys of 24 fingers, TBD measurements (medians and interquartile ranges) were G1, 0.95 mm (0.77–1.33); G2, 2.11 mm (1.78–2.33); G3, 2.28 mm (1.95–2.42); G4, 3.06 mm (2.79–3.28); and G5, 3.66 mm (3.55–4.76). These measurements and the numbers of samples in each group are provided in Table 1.

**TABLE 1:** Ultrasound TBD measurements.

Group	A2 pulley incision length	Sample size	D2	D3	D4	Total
	mm	n	Median (IQR)	Median (IQR)	Median (IQR)	Median (IQR)
						(Min-Max)
1	0	5	1.14 (0.95–1.33)	0.63 (0.49–0.77)	1.51 (1.51–1.51)	0.95 (0.77–1.33)
Intact tissue						(0.49–1.51)
2	5	6	1.88 (1.75–2)	2.06 (1.78–2.33)	2.44 (2.21–2.67)	2.11 (1.78–2.33)
Low-grade rupture						(1.75–2.67)
3	10	4	2.28 (2.23–2.33)	1.67 (1.67–1.67)	2.51 (2.51–2.51)	2.28 (1.95–2.42)
Medium-grade rupture						(1.67–2.51)
4	15	5	2.93 (2.79–3.06)	2.49 (2.29–2.49)	3.28 (3.28–3.28)	3.06 (2.79–3.28)
High-grade rupture						(2.49–3.28)
5	20	4	3.66 (3.62–3.69)	5.82 (5.82–5.82)	3.47 (3.47–3.47)	3.66 (3.55–4.76)
Complete rupture						(3.47–5.82)

D2, index digit; D3, middle digit; D4, annular digit; IQR, interquartile range; Min-Max, minimum–maximum.

**FIGURE 4:**
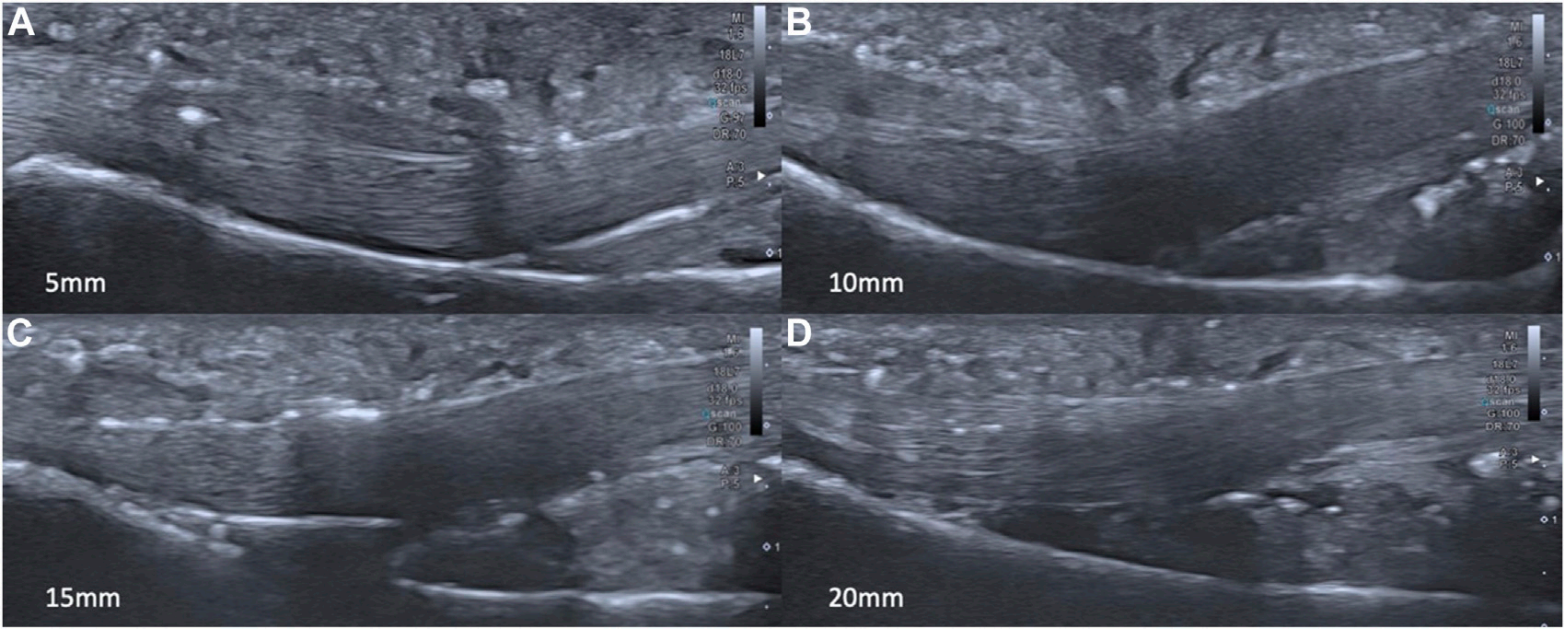
US TBD of each simulated tear size: G2 **(A)**; G3 **(B)**; G4 **(C)**; G5 **(D)**.

**FIGURE 5:**
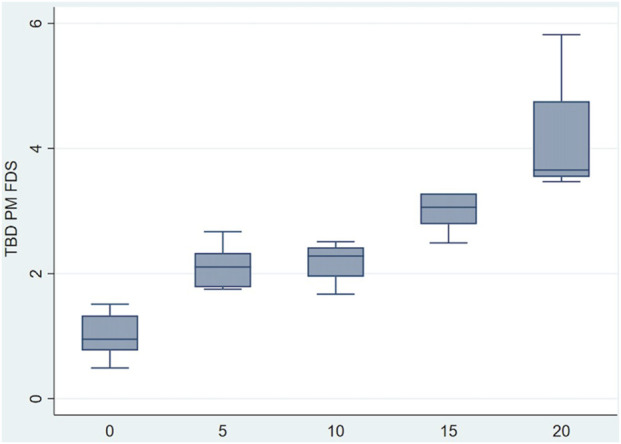
Boxplot of TBD values recorded for simulated different grades of ruptures.

The TBD values showed a significant increasing trend (*p* < 0.05): the larger the simulated pulley rupture, the larger the TBD (Figures 4, 5).

When comparing the TBD values recorded for the A2 pulleys among the groups, significant differences were found between the control non-torn pulleys (G1) and the simulated partial and complete pulley ruptures (vs. G2, *p* < 0.05; vs. G4 and G5, *p* < 0.05). In turn, TBD values were significantly different for the simulated partial and complete pulley ruptures (G2 vs. G5 *p* < 0.05; G3 vs. G5 *p* < 0.05). In contrast, no significant differences were found among the different partial rupture groups (G2 vs. G3 *p* = 1.00; G2 vs. G4 *p* = 0.201; G3 vs. G4 *p* = 0.469) or between the simulated high-grade partial and complete ruptures (G4 vs. G5, *p* = 0.055).

## 4 Discussion

This is only the second report to evaluate the effect on the TBD on US of variable length incisions of the A2 pulley simulating various degrees of partial rupture in a cadaver model. Our data support the notion that significant TBD differences exist between non-sectioned, partly sectioned, and fully sectioned pulleys (Leeflang and Coert, 2014). Inconsistent with the findings of Leeflang and Coert (2014), we found no significant differences in the TBD among different lengths of partial incisions. This difference could be related to the following technical differences. Leeflang and Coert (2014) progressively sectioned the same fingers whereas we chose to randomize our sample. In addition, pulley incision lengths differed, as we performed 5, 10, 15, and 20 mm incisions, whereas Leeflang and Coert (2014) incised the pulley in thirds. The ultrasound measurement method also varied. Leeflang and Coert (2014) recorded TBD measurements over a 5 mm section, we measured the TBD at the proximal phalanx’s midpoint as recommended by several researchers (Schöffl et al., 2006; Bassemir et al., 2015; Schöffl et al., 2018).

The TBD values of the sectioned A2 pulleys obtained here are compatible with those reported in two *in vivo* studies (Bodner et al., 1999; Klauser et al., 2002) and one cadaveric trial (Hauger et al., 2000). Our minimum and maximum TBD values of the partially sectioned A2 pulleys varied between 1.67 and 3.28 mm, which is close to the ranges reported in the two studies of 1.8–3 mm (Bodner et al., 1999) and 1–3.1 mm (Klauser et al., 2002). The difference was caused by the following: in these two studies, the sample consisted of traumatic partial ruptures in climbers, and the partial rupture size were not specified. Our results are consistent with those of Hauger et al. (2000): partial A2 pulley ruptures can be diagnosed by ultrasound as a significant TBD increase that is nevertheless lower than that of complete A2 ruptures. However, our data are not in agreement with the distances detected: for a 10 mm distal-to-proximal incision in the A2 pulley, the same as the incision in our group 3, the mean TBD was 1.4 mm and ranged from 0 to 2 mm (Hauger et al., 2000), which is much lower than our mean of 2.18 mm and range of 1.67–2.51 mm. This difference might be explained by the degree of force of flexor activation of the finger. Hauger et al. (2000) applied a 500 g traction force attached to the common flexor tendon of each finger; in this study, a traction force of 5 kg was applied. No consensus has been reached regarding the finger position and the optimal amount of activation or traction force in the ultrasound assessment protocol (Iruretagoiena-Urbieta et al., 2020b).

In the literature, debate is ongoing regarding the TBD cutoff that should be used to diagnose a partial A2 pulley rupture: >1.4 mm (Hauger et al., 2000), >1.5 mm (Klauser et al., 2002), <2 mm (Schöffl et al., 2003; Schöffl et al., 2018), or >2.2 mm (Bodner et al., 1999). The explanation for these differences could be that these values have not been related to a specific partial rupture size. As such, our distances fell between these limits but were always associated with different lengths of A2 pulley incisions, as we detected TBD values under 2 mm for 5 mm sections or above 2.2 mm for 10 mm sections. Consensus is also lacking regarding the anatomy landmark for TBD measurement (Iruretagoiena-Urbieta et al., 2020b), distal third of the proximal phalanx (Bowers et al., 1994; Klauser et al., 2002), or distal end of A2 pulley (Bodner et al., 1999) versus midpoint of the proximal phalanx (Schöffl and Schöffl, 2006). Therefore, the similarities with these values are not valid.

In contrast to the findings of others (Schöffl et al., 2003), we found partial ruptures of the A2 pulley with a TBD greater than 2 mm (in 66.6% of G2, 75% of G3, and 100% of G4). Accordingly, we think that this value cannot serve to directly diagnose a complete rupture of the A2 pulley. Conversely, rarely did we record a TBD > 3 mm for partial ruptures of small or medium ruptures (in 0% of G2 and G3, and 60% of G4), suggesting this value as a good cutoff for the diagnosis of large partial ruptures and especially, complete ruptures.

The clinical aim of detecting partial ruptures of the A2 pulley is to obtain a more accurate diagnosis to allow a precise classification of the injury degree (Schöffl and Schöffl, 2006; Lutter et al., 2021); hence, the return to climbing period can be estimated with increased accuracy. This will also help in the conservative treatment choice and to decide whether to use a thermoplastic ring (Schneeberger and Schweizer, 2016).

For a correct understanding of this section, caution should be exercised when comparing *in vivo* and *in vitro* specimens. This might distort the comparisons of TBD values among studies. The main limitations of the present study are its small sample size and the distortion due to the artifacts produced in the ultrasound images because of prior dissection. A possible solution to this problem may be taking ultrasound measurements in a water tank, but this would hinder the study of large numbers of fingers, and the visibility is higher using gel (Schöffl et al., 2018). The main limitation was that no statistically significant differences were found among different size partial rupture groups and future studies should measure TBD at more anatomic landmarks of the proximal phalanx to be more accurate for small partial rupture diagnosis. Another possible limitation could be that we did not use a fixation device to ensure finger position during US examinations, as performed in previous studies (Marco et al., 1998). However, we did monitor at all times finger joints position using a goniometer to ensure the accuracy of measurements. Further study is needed on a larger sample to confirm the TBD differences detected here between different sized partial ruptures and possible differences between the fingers. Further research is also needed with direct and indirect US manifestations to distinguish between high-grade partial ruptures and complete ruptures. Additional investigation could focus on setting subdivisions within grade II pulley ruptures to obtain more detailed information about treatment and time to recovery for each partial rupture size of A2 pulley. Furthermore, these results need to be compared with *in vivo* findings, despite the difficulty involved in finding control reference values.

The main conclusion of this study is that significant TBD differences were found between non-torn, simulated partial, and simulated complete rupture pulleys. This means that when partially sectioning the A2 pulley, clear separation is produced between the flexor tendons and the proximal phalanx even for incisions as short as 5 mm, representing one-third or even less of the total pulley length. The minimum TBD value for a partial rupture was 1.67 mm. Furthermore, the increase in TBD observed progressively increased the longer the pulley incision. The mean distance for the fingers examined was 2.11 (5 mm incision) to 3.66 mm (15 mm incision), which confirmed the capacity of ultrasound to diagnose small partial lesions. Additionally, for the different lengths of incisions, we did not find TDB values greater than 3 mm in low- or medium-grade partial ruptures (5 and 10 mm incision groups). This means that TBD values below 3 mm suggest a partial rupture, whereas values above this indicate a suspected complete lesion of the A2 pulley or a high-grade partial pulley rupture (15 mm incision). However, no significant differences were found among the 5, 10, and 15 mm simulated partial ruptures, which suggests that more research is needed.

## Data Availability

The original contributions presented in the study are included in the article/supplementary material, further inquiries can be directed to the corresponding author.
